# Normobaric and hyperbaric gas therapies in neurodegeneration: toward a multimodal protocol for early intervention

**DOI:** 10.4103/mgr.MEDGASRES-D-25-00162

**Published:** 2025-10-02

**Authors:** Natalia Marianna Kubiś, Lena Merchel, Maria Teper, Bartosz Palacz, Karol Seweryn Błąd

**Affiliations:** Faculty of Medicine, Jan Kochanowski University of Kielce, Kielce, Poland; Independent Public Health Care Institution of the Ministry of Interior Affairs and Administration in Kielce, Kielce, Poland; Central Teaching Hospital of the Medical University of Lodz, Lodz, Poland

Neurodegenerative diseases, such as Alzheimer’s disease (AD), Parkinson’s disease (PD), and vascular dementia, are among the most pressing medical and socioeconomic challenges facing aging populations worldwide. These conditions are characterized by gradual neuron loss, cognitive decline, motor dysfunction and growing dependence which cause significant quality of life-long depletion and poses challenges to families, carers and public health systems.^1^ Over 47 million people worldwide are estimated to suffer from dementia, with the likelihood of it increasing to 75 million in 2030 and 132 million by 2050 due to increased life expectancy.^2^ Despite ongoing pharmacological developments, the therapeutic efficacy of newer treatments like acetylcholinesterase inhibitors in AD and dopaminergic agents in PD remains limited, particularly in decelerating disease progression. In numerous instances, the drugs available only provide temporary relief and do not affect the underlying pathophysiological mechanisms, including mitochondrial dysfunction, neuroinflammation, and impaired neurovascular integrity.^1^ The lack of progress in disease-modifying trials has highlighted the need for innovative therapeutic methods that can target multiple pathological pathways simultaneously and intervene earlier in the disease progression. Medical gases have become a growing focus in this context as neuromodulatory and neuroprotective agents. Gases such as oxygen and hydrogen, when administered under normobaric (atmospheric pressure) or hyperbaric (elevated pressure) conditions, have the potential to cause complex effects on neural tissue.^1^ These include reducing reactive oxygen species, stabilizing the mitochondrial membrane potential, modulating pro- and anti-inflammatory cytokines, increasing cerebral perfusion, stimulating neurogenesis, and synaptic plasticity.^1^,^3^ Normobaric oxygen (NBO) therapy has been shown to enhance brain metabolism and improve outcomes in ischemic stroke and early cognitive impairment.^1^ Conversely, hyperbaric oxygen therapy (HBOT) by delivering high concentrations of oxygen under pressure, can profoundly increase oxygen diffusion into hypoxic tissues,^1^ offering benefits in traumatic brain injury, PD, and AD.^1^,^3^ Moreover, hydrogen and hydrogen sulfide are being researched for their anti-inflammatory, antioxidative, and anti-apoptotic properties in preclinical models of neurodegeneration.^1^,^4^ The idea suggests the development and clinical trials of a combined therapeutic protocol that employs both NBO and HBOT for various phases of neurodegenerative illness. By utilizing strategic timing, dosing, and combination with other therapies such as pharmaceuticals or lifestyle modifications, medical gas therapies may alter the treatment paradigm from reactive symptom management to proactive neuroprotection. The implementation of this technique could result in earlier intervention, enhanced therapy customization, and improved outcomes for patients with neurodegenerative diseases.^1^

**The promise of NBO therapy:** NBO therapy has demonstrated remarkable potential in modulating cerebral metabolism and improving outcomes for neurovascular and neurodegenerative diseases (**Figure 1**), as it involves breathing oxygen at one atmosphere pressure (1 ATA =101.325 kPa).^4^,^5^,^6^ Recent preclinical research has revealed that NBO can help reduce infarct volume, attenuate oxidative stress, and promote mitochondrial integrity after ischemic stroke. Cerebrovascular recanalization strategies can be supported by normobaric hyperoxia, as demonstrated by Li et al.,^6^ through clinical trials that support these findings. In this single-blind, sham-controlled trial normobaric hyperoxia as an addition to endovascular treatment in patients with acute ischemic stroke caused by large-vessel occlusion in the anterior circulation was associated with enhanced functional outcomes at 90 days without elevating the risk of adverse effects.^6^ NBO has demonstrated success in treating more than just stroke, including other psychiatric and cognitive disorders.^4^,^5^ According to a randomized double-blind, proof-of-concept trial conducted by Bloch et al.,^4^ NBO therapy may improve depression symptoms by increasing the rate of cerebral perfusion and decreasing inflammation. Due to its simplicity, non-invasiveness, and safety, it represents a potentially promising and appealing treatment option for depression.^4^ In the same way, Wang et al.^5^ demonstrated its effectiveness in treating mild cognitive impairment through neurorehabilitation, which resulted in improvements in memory, executive function, and quality of life. This indicates that NBO could offer valuable opportunities for neurorehabilitation and motor learning strategies could offer valuable opportunities for neural recovery therapy and motor learning strategies.^5^ In clinical settings, NBO can be delivered via nasal cannula or face mask and is often available in emergency departments and stroke units.^4^,^6^ NBO is a valuable addition to early-stage intervention approaches due to its ease of use, easy administration, and safety profile.^1^,^6^ Nevertheless, there are uncertainties regarding the most effective dosing regimens, therapy duration, and patient selection criteria. Future research should focus on identifying biological markers or imaging correlates that predict response to NBO, making it easier to design personalized interventions.^1^

**Figure 1:**
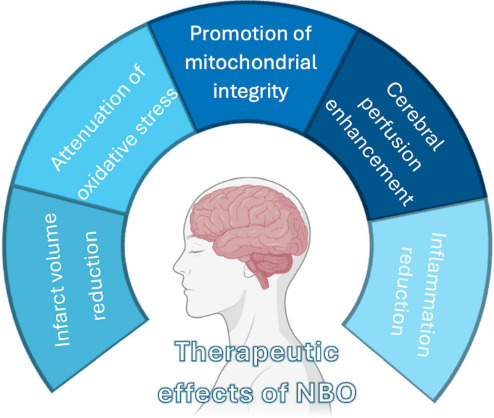
Schematic representation of the therapeutic effects of normobaric oxygen (NBO) therapy.^4^,^6^ Created with BioRender.com.

**Advances in HBOT:** HBOT involves breathing 100% oxygen at pressures greater than atmospheric pressure, typically 1.5 to 3.0 ATA.^1^ This improves oxygen levels in blood plasma, enhances oxygen delivery to tissues, and exerts a variety of systemic influences (**Figure 2**).^1^,^3^ HBOT has demonstrated potential in various areas of neurodegeneration research.^1^ According to Bu et al.,^7^ motor symptoms, sleep quality, and daily functioning were significantly improved in patients with PD treated with HBOT. Moreover, it shows the ability to slow the advancement of PD through various neuroprotective effect.^7^ Long-term HBOT in AD patients was demonstrated by Chen et al.^8^ to result in cognitive and functional improvements as well. Even though it remains unclear whether the therapeutic effect of HBOT is directly affecting brain functions or indirectly influencing the effectiveness of drugs administered to AD patients.^8^ The benefits are thought to be derived from enhanced mitochondrial function, decreased apoptosis, and modulation of inflammatory cytokines while stimulating neurogenesis and angiogenesis.^1^,^3^ In a notable study, Zilberman-Itskovich et al.^9^ found that HBOT significantly reversed cognitive decline in post-COVID patients, a population increasingly recognized to be at risk for neuroinflammatory and neurodegenerative complications. These findings suggest that HBOT can provide more than just relief from symptoms but also have a role in upstream pathological processes. Moreover, given the association between mitochondrial dysfunction, oxidative stress, and various diseases, increasing antioxidant activity may be one of the key mechanisms by which HBOT exerts its clinical effects. In addition to this, enhanced mental and emotional abilities may result from the brain’s improved adaptability and greater blood circulation in key regions involved in these functions.^9^ Nevertheless, HBOT is not without risks or limitations. Common side effects include middle ear barotrauma, temporary myopia, and claustrophobia. Risks of serious complications, such as seizures caused by oxygen toxicity, should be considered, especially in vulnerable elderly patients.^1^

**Figure 2:**
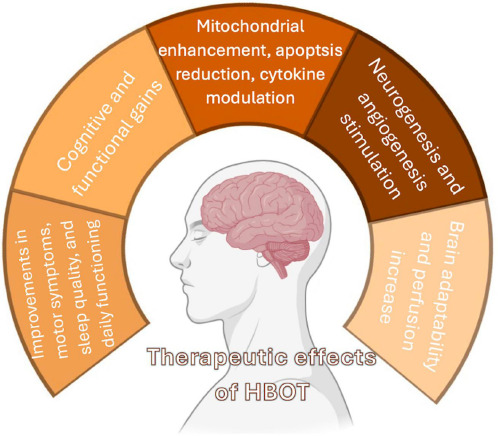
Schematic representation of the therapeutic effects of hyperbaric oxygen therapy (HBOT).^1^,^3^,^7^,^8^,^9^ Created with BioRender.com.

**Toward a multimodal protocol:** The combination of NBO and HBOT in a single, specific therapy may offer the highest potential outcomes due to their mutually beneficial mechanisms. In early or prodromal stages of neurodegenerative disease, NBO could be used regularly to enhance oxygenation, protect vulnerable neurons, and promote metabolic resilience. Regeneration, neuroplasticity, and microvascular remodeling could be promoted by introducing periodic HBOT as the disease progresses.^1^ Moreover, such gas-based interventions should not be viewed in isolation. The use of pharmacologic agents (e.g., cholinesterase inhibitors, anti-inflammatory drugs), physical rehabilitation, and cognitive therapy could lead to synergistic effects.^1^,^10^ Tailoring this multimodal strategy based on patient-specific biomarkers, genetic predispositions, and imaging findings could further enhance efficacy.^1^ Recent research also supports the integration of gases such as H_2_. In preclinical models of neurodegeneration, these agents have demonstrated antioxidants, anti-apoptotic and anti-inflammatory properties. Hydrogen inhalation as a neuromodulatory therapy is being researched for its potential impact on neurodegenerative diseases. According to preclinical studies, it may decrease glial activation, which contributes to chronic inflammation and neuronal dysfunction. By stabilizing neural circuits and facilitating synaptic communication, hydrogen may have the potential to preserve brain function. Its antioxidant and anti-inflammatory properties are likely responsible for these effects, making it a supplementary approach to conventional treatments.^11^ With research ongoing, these gases could potentially serve as essential components within the wider multimodal framework.^1^ In practice, a suggested approach may involve initial screening with neuroimaging and blood-based biomarkers of neuroinflammation,^12^ conducting baseline cognitive and functional assessments,^9^ administering NBO sessions,^4^,^5^ evaluating clinical response, targeted HBOT sessions in responders or at signs of progression^8^,^9^ and maintenance with adjunctive hydrogen therapy and lifestyle interventions.^1^ Such a theoretical framework shows how gas-based interventions could be useful for monitoring therapy effectiveness in real time and develop personalized treatment plans. Large-scale, randomized, controlled trials are not available for many indications, which limits clinical adoption. Additionally, logistical barriers, such as the limited availability of HBOT chamber, the requirement for medical supervision and the high associated costs, pose significant barriers to its widespread implementation and accessibility for many patients. Advancing this field will require collaborative multicenter studies.^1^ There is also a need to examine interactions between gas therapies and concurrent medications, as well a long-term safety and adherence study in different patient populations.^1^,^11^

**Conclusion:** The adoption of a stage-specific protocol for NBO and HBOT could significantly impact early intervention and disease change in neurodegenerative disorders. Their complementary mechanisms, which include metabolic support and neuroregeneration, make them promising candidates for the shift from reactive to proactive care. Despite the challenges faced, the inclusion of these therapies in multimodal, personalized treatment plans could signal a significant advancement in the field of neurodegeneration management. Considering the dynamic and stage-dependent nature of neurodegenerative processes, a tailored approach to NBO and HBOT that aligns with specific disease stages may optimize therapeutic efficacy. Early intervention with appropriately timed oxygen therapy could enhance neuroprotection, while later-stage strategies might aim to preserve residual function and reduce progression. While the discussed interventions hold considerable promise, their clinical application requires validation through extensive, controlled studies to fully establish their therapeutic value. Future studies should focus on identifying the optimal therapeutic window for therapy, especially during prodromal stages when neuronal damage may still be reversible.

## References

[1] Barata P, Camacho O, Lima CG, Pereira AC (2024). The role of hyperbaric oxygen therapy in neuroregeneration and neuroprotection: a review. Cureus.

[2] World Health Organization Global action plan on the public health response to dementia. https://iris.who.int/bitstream/handle/10665/259615/9789241513487-eng.pdf?sequence=1.

[3] Schottlender N, Gottfried I, Ashery U (2021). Hyperbaric oxygen treatment: effects on mitochondrial function and oxidative stress. Biomolecules.

[4] Bloch Y, Belmaker RH, Shvartzman P (2021). Normobaric oxygen treatment for mild-to-moderate depression: a randomized, double-blind, proof-of-concept trial. Sci Rep.

[5] Wang Z, Spielmann G, Johannsen N, Greenway F, Irving BA, Dalecki M (2023). Boost your brain: a simple 100% normobaric oxygen treatment improves human motor learning processes. Front Neurosci.

[6] Li W, Lan J, Wei M (2025). Normobaric hyperoxia combined with endovascular treatment for acute ischaemic stroke in China (OPENS-2 trial): a multicentre, randomised, single-blind, sham-controlled trial. Lancet.

[7] Bu S, Liu W, Sheng X, Jin L, Zhao Q (2025). Hyperbaric oxygen therapy improves motor symptoms, sleep, and cognitive dysfunctions in Parkinson’s disease. Dement Geriatr Cogn Disord.

[8] Chen J, Zhang F, Zhao L (2020). Hyperbaric oxygen ameliorates cognitive impairment in patients with Alzheimer’s disease and amnestic mild cognitive impairment. Alzheimers Dement (N Y).

[9] Zilberman-Itskovich S, Catalogna M, Sasson E (2022). Hyperbaric oxygen therapy improves neurocognitive functions and symptoms of post-COVID condition: randomized controlled trial. Sci Rep.

[10] Livingston G, Huntley J, Liu KY (2024). Dementia prevention, intervention, and care: 2024 report of the Lancet standing Commission. Lancet.

[11] Wang F, Zhang G, Zhai Q (2025). Role and mechanism of molecular hydrogen in the treatment of Parkinson’s diseases. Front Neurosci.

[12] Garcia-Escobar G, Manero RM, Fernández-Lebrero A (2024). Blood biomarkers of Alzheimer’s disease and cognition: a literature review. Biomolecules.

